# Effects of PI3K inhibitor NVP-BKM120 on overcoming drug resistance and eliminating cancer stem cells in human breast cancer cells

**DOI:** 10.1038/cddis.2015.363

**Published:** 2015-12-17

**Authors:** Y Hu, R Guo, J Wei, Y Zhou, W Ji, J Liu, X Zhi, J Zhang

**Affiliations:** 1 The 3rd Department of Breast Cancer, China Tianjin Breast Cancer Prevention, Treatment and Research Center, Tianjin Medical University Cancer Institute and Hospital, National Clinical Research Center of Cancer, Tianjin, China; 2 Key Laboratory of Breast Cancer Prevention and Therapy of Ministry of Education, Tianjin, China

## Abstract

The multidrug resistance (MDR) phenotype often accompanies activation of the phosphatidylinositol 3-kinase (PI3K)/AKT pathway, which renders a survival signal to withstand cytotoxic anticancer drugs and enhances cancer stem cell (CSC) characteristics. As a result, PI3K/AKT-blocking approaches have been proposed as antineoplastic strategies, and inhibitors of PI3K/AKT are currently being trailed clinically in breast cancer patients. However, the effects of PI3K inhibitors on MDR breast cancers have not yet been elucidated. In the present study, the tumorigenic properties of three MDR breast cancer cell lines to a selective inhibitor of PI3K, NVP-BKM120 (BKM120), were assessed. We found that BKM120 showed a significant cytotoxic activity on MDR breast cancer cells both *in vitro* and *in vivo*. When doxorubicin (DOX) was combined with BKM120, strong synergistic antiproliferative effect was observed. BKM120 activity induced the blockage of PI3K/AKT signaling and NF-*κ*B expression, which in turn led to activate caspase-3/7 and caspase-9 and changed the expression of several apoptosis-related gene expression. Furthermore, BKM120 effectively eliminated CSC subpopulation and reduced sphere formation of these drug-resistant cells. Our findings indicate that BKM120 partially overcomes the MDR phenotype in chemoresistant breast cancer through cell apoptosis induction and CSC abolishing, which appears to be mediated by the inhibition of the PI3K/AKT/NF-*κ*B axis. This offers a strong rationale to explore the therapeutic strategy of using BKM120 alone or in combination for chemotherapy-nonresponsive breast cancer patients.

Multidrug resistance (MDR) remains a major cause for failure of chemotherapy-based treatment of breast cancer, in which cells become refractory to many structurally and functionally unrelated chemotherapeutic drugs.^1^ Overexpression of P-glycoprotein (P-gp), a member of the ATP-binding cassette (ABC) transporter family encoded by the *mdr-1* gene, represents one of the principal mechanisms that contribute to the MDR phenotype.^2^ However, many other mechanisms contribute simultaneously to the MDR phenotype, which may affect drug absorption, distribution and metabolism, thus modulating the efficacy of chemotherapeutic agents consequently.^3, 4^ Growing evidence supports the notion that a subset of cancer cells, with self-renewal and differentiation features, are the cancer stem cells (CSCs) thought to be responsible for resistance to chemotherapy.^5^ CSCs seem to be protected against chemotherapeutic agents by means of different mechanisms, such as robust proficiency of DNA damage repair, overexpression of ABC transporters, abnormal activation of numerous signaling pathways, including phosphatidylinositol 3-kinase (PI3K)/AKT, Notch, Hedgehog and Wnt pathways.^6, 7, 8^ On the other hand, the CSC fraction is probably enriched after chemotherapy, as demonstrated by the increased expression of stemness markers in patients who are receiving primary systematic therapy.^9^

The activation of the PI3K/AKT pathway is frequently implicated in resistance to anticancer therapies. Once activated, AKT can phosphorylate multiple substrates and downstream effectors, such as mTOR family, caspase family, cell cycle protein family and nuclear factor-*κ*B (NF-*κ*B), which contribute collectively to promote cell proliferation, survival, metastasis and chemoresistance.^10, 11, 12^ As this signaling cascade has a central role in human breast cancer, development of novel strategies to overcome resistance and eliminate CSC by targeting the PI3K/AKT pathway is apparently warranted.^13^

NVP-BKM120 (referred hereafter as BKM120) is a potent and highly selective pan-class I PI3K inhibitor, which belongs to the 2,6-dimorpholino pyrimidine derivatives.^14^ It selectively inhibits wild type and mutant PI3K p110 *α*, *β*, *δ*, *γ* isoforms and exerts a strong antiproliferative effect to induce apoptosis in several cancers by specifically inhibiting the PI3K/AKT signaling pathway.^15, 16, 17^ Phase I clinical trials show that overall BKM120 is well tolerated in several solid tumors, and Phase II clinical trials are ongoing.^17^ Several recent reports also emphasized the enhanced antitumor effects in mouse models when BKM120 was co-treated with inhibitors of other signaling pathways.^18, 19, 20^

In this study, we analyzed, for the first time, the efficacy of BKM120 in several MDR breast cancer cell lines with which the MDR phenotype is induced by different molecular mechanisms. BKM120 exerted potent efficacy of apoptosis promoting as well as CSCs eliminating through inhibiting the PI3K/AKT/NF-*κ*B cascade *in vitro* and *in vivo*. In addition, BKM120 synergized with DOX, a common chemotherapeutic agent of breast cancer. Here we demonstrate the potential of BKM120 in overcoming chemotherapy resistance in breast cancer.

## Results

### PI3K inhibitor BKM120 shows potent cytotoxicity against both sensitive and MDR breast cancer cell lines

**Figure 1:**
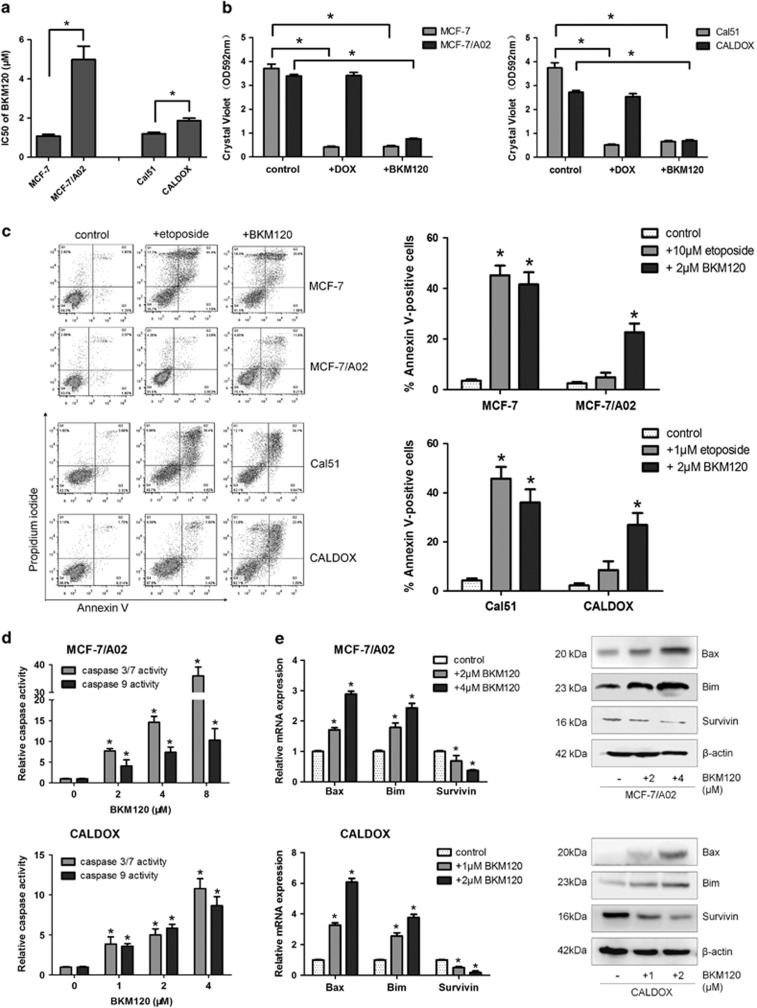
BKM120 is cytotoxic to MDR breast cancer cells by activating apoptosis. (**a**) IC_50_ values of BKM120 of two pairs of human breast cancer cell lines and their MDR sublines. (**b**) Cells were treated with doxorubicin (3 *μ*M for MCF-7 and MCF-7/A02, 0.2 *μ*M for Cal51 and CALDOX) and BKM120 (2 *μ*M for all cell lines) for 7 days, and the cells were stained with crystal violet. Dye was solubilized and the optical density at 592 nm was measured. (**c**) Cells were treated with etoposide (10 *μ*M for MCF-7 and MCF-7/A02, 1 *μ*M for Cal51 and CALDOX) and BKM120 (2 *μ*M for all cell lines) for 48 h. Annexin V/PI staining was detected with flow cytometry. Representative plots of three independent experiments are shown. Quantitative data show the average percentage of annexin V-positive cells (both in early apoptosis, lower right quadrant and late apoptosis, upper right quadrant) of three independent experiments (right panel). (**d**) Caspase-3/7 and caspase-9 activities in MCF-7/A02 (upper histograms) and CALDOX cells (lower histograms) after BKM120 treatment. (**e**) Fold changes of Bax, Bim and Survivin expression levels in MCF-7/A02 and CALDOX cells determined using RT-qPCR (left panel) and western blot (right panel) after BKM120 treatment at various concentrations for 48 h. Numerical data are presented as mean±S.D. of three independent experiments. *P<0.05

The MDR breast cancer cell lines MCF-7/A02 and CALDOX were derived from chemosensitive cell lines MCF-7 and Cal51, respectively. The MDR phenotype of these derived cell lines was manifested by their cross-resistance to a wide range of structurally and functionally unrelated drugs (Supplementary Tables S1 and S2). BKM120 inhibited growth of chemosensitive and chemoresistant breast cancer cells in a dose-dependent manner (Supplementary Figure S1A); the IC50 assay results revealed that two drug-resistant cell lines exhibited only 4.64- and 1.56-fold resistance to BKM120, respectively, compared with their parental chemosensitive counterparts (Figure 1a). Similar results were also observed in another pair of breast cancer cell lines, the relatively sensitive MTMEC cell line and its DOX-resistant derivative MD60 cell line (Supplementary Figures S2A and B). BKM120 appeared to be more effective in eliminating drug-resistant cells than chemotherapeutic drugs. To further evaluate the cytotoxic effect of BKM120 on chemosensitive and chemoresistant breast cancer cells, the cells were treated with either DOX or BKM120. Crystal violet staining was used to determine the cell mass 1 week after treatment. As expected, the chemoresistant cells robustly defied DOX, whereas the same concentration of DOX eradicated most drug-naive cells. However, BKM120 significantly decreased the capacity of MDR cells to survive after 1-week treatment (reduction ranging from 72.4 to 77.7%, Figure 1b and Supplementary Figure S1B). These results demonstrate that BKM120 is a potent cytotoxic agent to MDR breast cancer cells.

### BKM120 promotes cell apoptosis, induces caspase activity and modulates apoptosis-related genes

Next, we asked whether the cytotoxic effect of BKM120 on chemoresistant breast cancer cells is mediated by promoting cell apoptosis. As DOX can be excited at 488 nm by blue excitation laser, with emission at ~595 nm,^21^ thereby interfering with the results of the Annexin V/propidium iodide (PI) staining assay, we used etoposide, another widely used drug known to induce cell apoptosis^22, 23^ as a positive control. As illustrated in Figure 1c, 10 *μ*M etoposide showed similar apoptosis-inducing effect as 2 *μ*M BKM120 did in MCF-7 cells, but failed to do so in MCF-7/A02 cells. After 2*-μ*M BKM120 treatment, the percentage of apoptotic MCF-7/A02 cells increased remarkably. A similar effect of BKM120 treatment on promoting apoptosis was also observed in CALDOX cells. In accordance with the Annexin V/PI analysis, BKM120 strongly induced caspase-3/7 and caspase-9 activities in MCF-7/A02 and CALDOX cells, respectively (Figure 1d), which was also confirmed in MD60 cells (Supplementary Figure S2C). As Supplementary Figure S1D shows, the pancaspase inhibitor z-VAD-fmk was able to abrogate Caspase-3/7 and Caspase 9 activities mediated by BKM120 intensively in MCF-7/A02 and CALDOX cells, confirming the specificity of the observed effects. Cells were untreated or pretreated with z-VAD-fmk for 4 h, followed by treatment with BKM120, and were subsequently analyzed for cell viability by MTT assay and cell apoptosis by Annexin V/PI staining assays. z-VAD-fmk reduced the percentage of Annexin V-positive cells (both PI low, early apoptotic and PI high, late apoptotic/necrotic) upon BKM120 treatment. In addition, there was almost a 2.6-fold shift in the IC_5__0_ for MCF-7/A02 cells and a 3.9-fold shift in the IC50 for CALDOX. These data demonstrate the specific induction of cell death by BKM120 through the activation of the apoptotic cascade (Supplementary Figure S1E and F). As the induction of apoptotic cell death could be partly due to alteration of apoptosis-related genes, we evaluated the mRNA levels of several survival genes, including *Bcl-2*, *Bcl-xl*, *Survivin* and *Mcl-1*, as well as apoptotic genes, *Bim* and *Bax*. Reverse transcription quantitative real-time-PCR (RT-qPCR) and western blot results revealed that BKM120 repressed pro-survival gene *Survivin* expression and upregulated pro-apoptotic genes *Bim* and *Bax* expression in MDR cells (Figure 1e), although the expressions of *Bcl-2*, *Bcl-xl* and *Mcl-1* were not changed (Supplementary Figure S1C).

**Figure 2:**
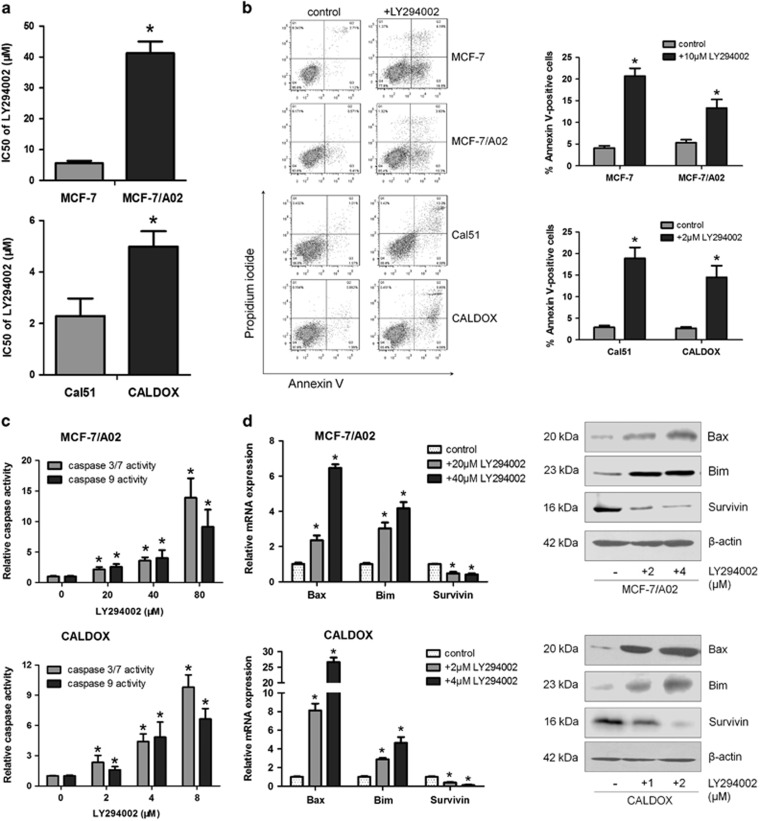
Blocking the PI3K/Akt pathway by LY294002 induces apoptosis in MDR breast cancer cells. (**a**) IC_50_ value of LY294002 in MCF-7 and MCF-7/A02 (upper panel), Cal51 and CALDOX (lower panel). (**b**) Cells were treated with LY294002 (10 *μ*M for MCF-7 and MCF-7/A02, 2 *μ*M for Cal51 and CALDOX) for 48 h. Annexin V/PI staining was detected using flow cytometry. Representative plots of three independent experiments are shown. Quantitative data show the average percentage of annexin V-positive cells (both in early apoptosis, lower right quadrant and late apoptosis, upper right quadrant) of three independent experiments (right panel). (**c**) Caspase-3/7 and caspase-9 activities of MCF-7/A02 (upper histograms) and CALDOX (lower histograms) after LY294002 treatment. (**d**) Fold changes of Bax, Bim and Survivin expression levels determined using RT-qPCR (left panel) and western blot (right panel) in MCF-7/A02 and CALDOX after LY294002 treatment with various concentrations for 48 h. Numerical data are presented as mean±S.D. of three independent replicates. *P<0.05

To further confirm that promoting effect of BKM120 on apoptosis is specifically mediated by PI3K/AKT inhibition, chemoresistant breast cancer cells were treated with LY294002, another well-characterized selective PI3K/Akt inhibitor. Similar to BKM120, the IC_50_ values of LY294002 in MCF-7/A02 and CALDOX cells are only 7.38 and 2.18 times greater than those in MCF-7 and Cal51 cells, respectively (Figure 2a). LY294002 significantly induced cell apoptosis and activated caspases in MCF-7/A02 and CALDOX cells (Figure 2b and c). In addition, LY294002 treatment also enhanced Bax and Bim expression and reduced Survivin mRNA and protein levels (Figure 2d). Thus, attenuating PI3K/AKT signaling appears to be an important pathway to induce chemoresistant breast cancer cell apoptosis.

### BKM120 induces cytotoxicity through blocking the PI3K/AKT/NF-*κ*B signaling pathway

**Figure 3:**
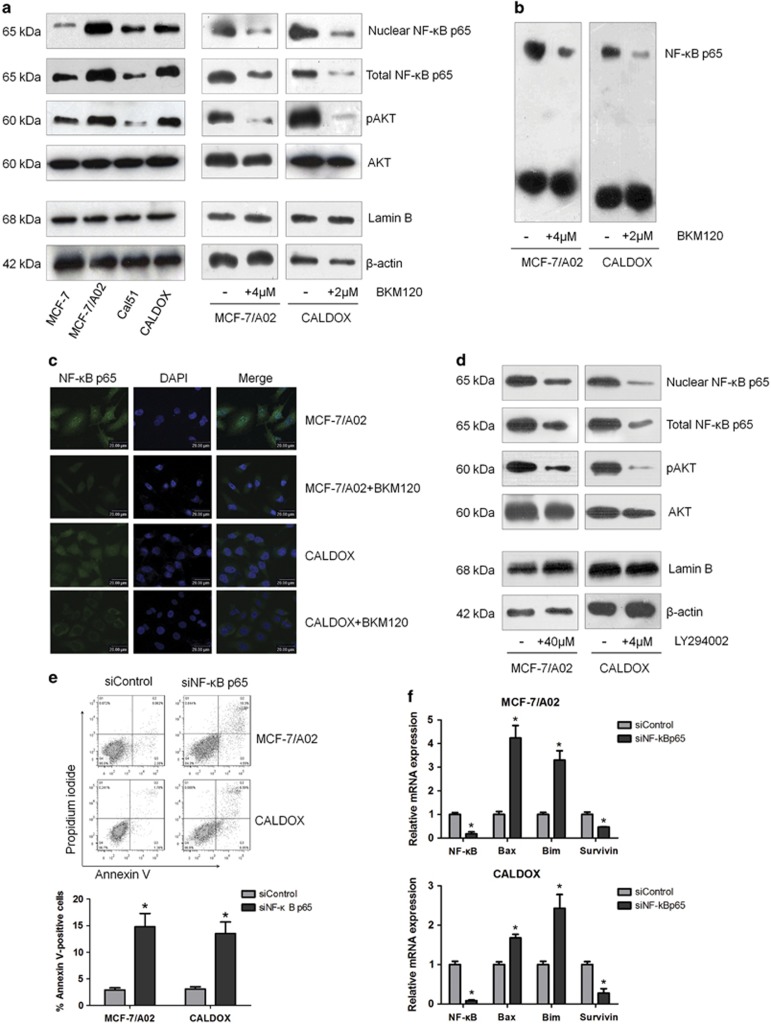
The PI3K/AKT pathway inhibitor induces apoptosis through suppressing NF-*ĸ*B activity. (**a**) Western blots show BKM120 downregulating pAKT, nuclear NF-*κ*B p65 and total NF-*κ*B p65 in MDR and their parental cells. *β*-actin was used as a loading control for pAKT, AKT and total NF-*κ*B p65. Lamin B was used as a loading control for nuclear NF-*κ*B p65. (**b**) EMSA results show that BKM120 treatments (4 *μ*M for MCF-7/A02 and 2 *μ*M for CALDOX) decreased NF-*κ*B DNA-binding activity in MDR cells. (**c**) Immunofluorescence staining of NF-*κ*B p65 in MDR cells treated with or without BKM120 (4 *μ*M for MCF-7/A02 and 2 *μ*M for CALDOX) for 48 h. (**d**) Western blots show LY294002 treatments (40 *μ*M for MCF-7/A02 and 4 *μ*M for CALDOX) downregulating pAKT, nuclear NF-*κ*B p65 and total NF-*κ*B p65 in MDR cells. (**e**) MCF-7/A02 and CALDOX cells were transiently transfected with NF-*κ*B p65 siRNA (siNF-*κ*B p65) or scrambled siRNA (siControl). Three days after transfection, cells were stained with Annexin V/PI and cell death was quantified using flow cytometry. Representative plots of three independent experiments are shown. Quantitative data show the average percentage of annexin V-positive cells (both in early apoptosis, lower right quadrant, and late apoptosis, upper right quadrant) of three independent experiments (lower panel). (**f**) Fold changes of NF-*κ*B p65, Bax, Bim and Survivin mRNA levels detected using RT-qPCR in MDR cells after NF-*κ*B p65 siRNA transfection for 72 h. Numerical data are presented as mean±S.D. of three independent replicates. *P<0.05

The PI3K/AKT signaling pathway is frequently dysregulated in human cancer and has been implicated in the development of resistance to standard anticancer therapies.^24, 25, 26^ It is known that chemoresistant MCF-7/A02 cells are mainly mediated by increased P-gp expression, whereas the chemoresistance in CALDOX cells is due to the loss of expression of TOP2A, rather than overexpression of membrane transporters.^27, 28^ Although the leading causes of MDR phenotype are quite different, the PI3K/AKT signaling cascade in these two MDR cell lines was found to be overactivated compared with their parental cell lines (Figure 3a). Our data indicated that induction of cell apoptosis by BKM120 in both MDR cancer cell lines was associated with a significant reduction in AKT phosphorylation, suggesting a decrease in PI3K activity (Figure 3a).

As PI3K/AKT signaling has a pivotal role through regulating diverse downstream effectors, we sought to determine the potential downstream effector that involves in BKM120-induced apoptosis in MDR cells. The crosstalk between NF-*κ*B activity and PI3K/AKT pathway has been shown in various cancers, and constitutive activation of NF-*κ*B induced by the PI3K/AKT pathway may have a major role in the development of chemoresistance.^29, 30^ Our western blot results revealed that MDR cells contained higher NF-*κ*B p65 protein levels in whole-cell extracts as well as in the nuclear compartment, where NF-*κ*B p65 exerts its transcriptional activity (Figure 3a). Therefore, we speculated that BKM120 exerts its apoptotic induction through downregulating the PI3K/AKT/NF-*κ*B cascade in MDR breast cancer cells. To address this issue, we examined mRNA and protein levels (Figure 3a and Supplementary Figure S3A), cellular localization (Figure 3a and c) and DNA-binding activity (Figure 3b) of the NF-*κ*B p65 subunit in MCF-7/A02 and CALDOX cells. The results showed that BKM120 significantly decreased NF-*κ*B p65 expression and its DNA-binding activity (Figure 3a–c and Supplementary Figure S3A). LY294002 also displayed a suppressive effect on NF-*κ*B p65 expression similar to what was observed in BKM120 treatment in these MDR cells (Figure 3d and Supplementary Figure S3B).

To ascertain whether the downregulation of NF-*κ*B activity influenced apoptosis-related gene expression, we used a small interfering RNA (siRNA) approach to knockdown NF-*κ*B p65. Figure 3e and f showed that NF-*κ*B p65 siRNA transfection significantly reduced NF-*κ*B p65 and Survivin mRNA levels, increased Bim and Bax expression and concomitantly led to cell apoptosis when compared with scramble siRNA-transfected cells. Taken together, these results demonstrate that PI3K/AKT inhibitor BKM120 induces MDR breast cancer cell apoptosis through suppressing NF-*κ*B activity.

### BKM120 eliminates stem cell subpopulation of MDR breast cancer cells

**Figure 4:**
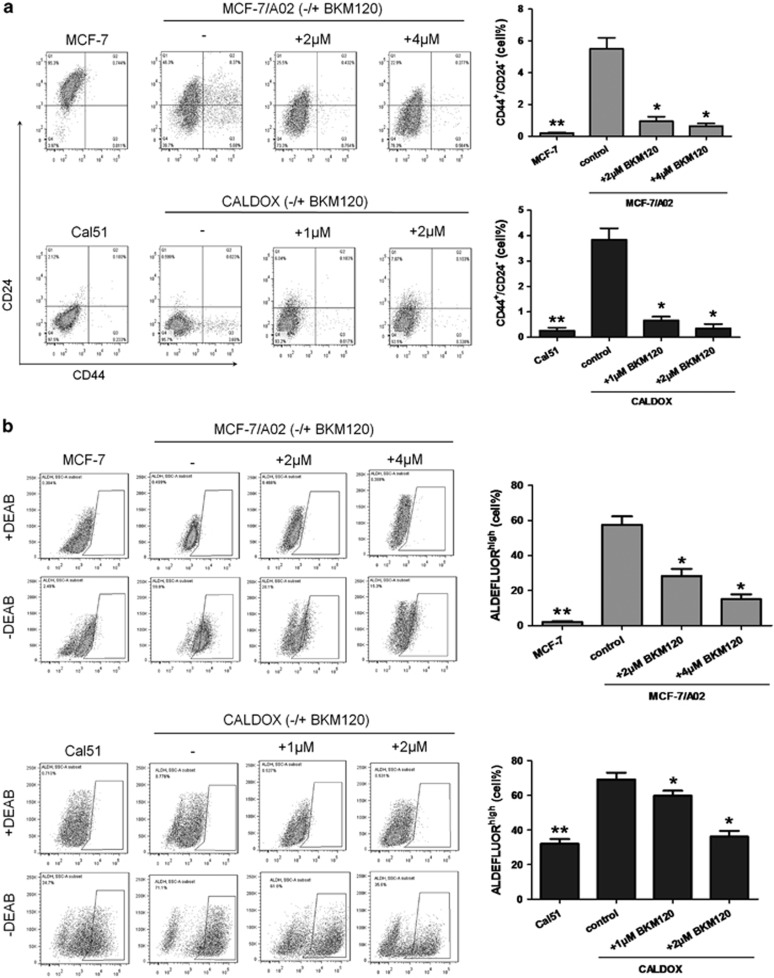

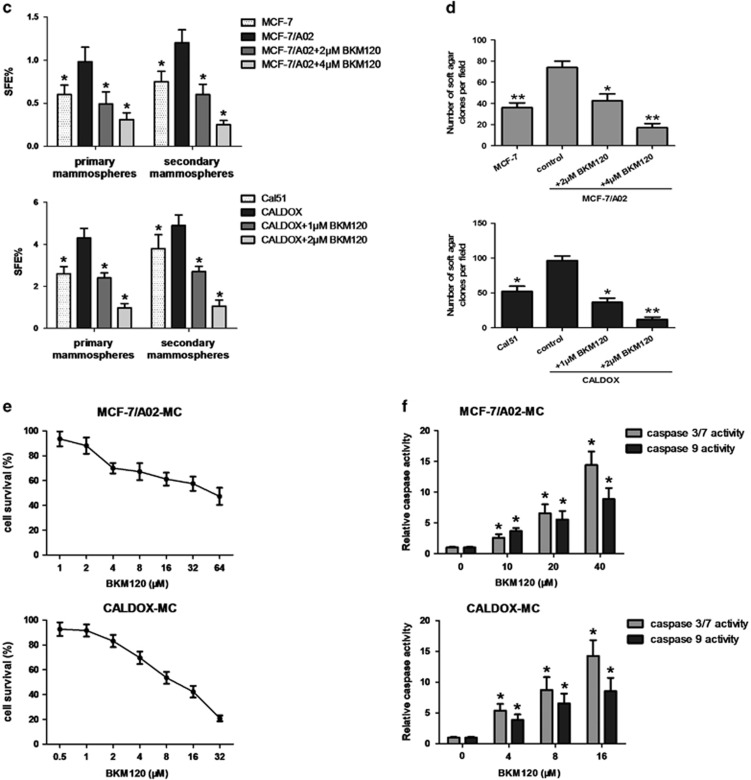
The inhibitory effects of BKM120 on SC in MDR breast cancer cells. (**a**) Flow cytometry plots for CD44 and CD24 of MDR and their parental cells. Cells in Q3 (CD44^high^CD24^low^) are associated with SC subpopulation. Quantitative data show the average percentage of CD44^high^CD24^low^ cells (right panel). (**b**) Flow cytometry analysis of ALDH activity. Cells treated with or without BKM120 for 48 h were assayed with an Aldefluor assay kit in the presence and absence of the ALDH inhibitor diethylaminobenzaldehyde (DEAB). Gating in the control was set up to a maximum of 1% of cells. Representative plots of at least three independent experiments are shown. Quantitative data show the average percentage of ALDH^high^ cells (right panel). (**c**) Primary and secondary mammosphere-forming efficacies of MDR and their parental cells. SFE was calculated as the number of spheres formed in 10 days divided by the original number of single cells seeded and expressed as percentage. Bars represent the mean percentage of mammospheres. (**d**) Anchorage independence was determined by the formation of clones in soft agar cultured for 3 weeks. Histogram data represent the average number of colonies counted in randomly chosen five visual fields under the microscope (magnification × 40). (**e**) Dose–response curves were used to calculate the IC_50_ of BKM120 for MCs of MCF-7/A02 cells (upper) and CALDOX cells (lower). (**f**) Caspase-3/7 and caspase-9 activities of MCF-7/A02-MC (upper histograms) and CALDOX-MC (lower histograms) after BKM120 treatment. Numerical data are presented as mean±S.D. of three independent experiments. *P<0.05, **P<0.01

Drug-resistant cells have been proposed to arise from the selection of a small population of cells with stem-like properties.^5^ BKM120 has been shown to have robust anticancer properties in MDR breast cancer cells; therefore, we then asked whether BKM120 could also eliminate stem cell population of MDR cells. First, we tested the effect of BKM120 on the proportion of stem-like cell (SC) population in chemoresistant breast cancer total cells (TCs). We analyzed the expression of CD44 and CD24, two cell-surface markers whose expression in the CD44^high^/CD24^low^ configuration is associated with breast SCs.^31^ Emergence of MDR was indeed accompanied by an increase in the percentage of CD44^high^/CD24^low^ cells (Figure 4a). As expected, the percentage of CD44^high^/CD24^low^ cells was reduced in MCF-7/A02 and CALDOX cells after BKM120 treatment in a range of doses lower than IC_50_ (Figure 4a). We then analyzed aldehyde dehydrogenase (ALDH) activity, another important marker of SCs.^32^ Comparing MCF-7 and Cal51 cells, both MCF-7/A02 and CALDOX cell lines were composed of a much higher population of cells exhibiting ALDH activity (Figure 4b), and the ALDH activity in these MDR cell lines were remarkably inhibited after exposure to BKM120 in the doses lower than IC_50_ (Figure 4b). Similar effect of BKM120 was observed in MD60 cells (Supplementary Figure S2E).

Breast SCs exhibit other stem-like properties, including the ability to survive and grow as spheres or colonies under certain conditions, such as low attachment plates and soft agar medium.^33^ First, we observed that MCF-7/A02 and CALDOX cells indeed formed higher numbers of spheres and clones than their parental cell lines (Figure 4c and d). Second, BKM120 eliminated MCF-7/A02 and CALDOX cells' sphere-forming efficacy (SFE), as well as the ability to produce colonies (Figure 4c and d and Supplementary Figure S4A and B). Third, primary mammospheres from each cell line were enzymatically dispersed to single cells and assayed for their ability to form secondary mammospheres. BKM120 treatment maintained SFE inhibition in the second passage of both MCF-7/A02 and CALDOX cell lines (Figure 4c).

As the mammosphere technique has been proven for enriching the highly tumorigenic SCs,^34, 35^ the mammosphere cells (MCs) from MCF-7/A02 and CALDOX cells were obtained through sphere-forming assay as mentioned above. Comparing with TCs, MC has more CD44^high^/CD24^low^ subpopulation and ALDH1^high^ subpopulation analyzed using flow cytometry (Supplementary Figure S5). As the MTT assay results show, DOX and etoposide were more cytotoxic in chemoresistant TCs than in their MCs. MCF-7/A02 and CALDOX MCs exhibited 68- and 47-fold greater resistance to DOX than MCF-7/A02 and CALDOX TCs, respectively (Table 1). In addition, MCF-7/A02 and CALDOX MCs exhibited 42- and 18-fold greater resistance to etoposide than MCF-7/A02 and CALDOX TCs, respectively (Table 1). BKM120 also displayed a cytotoxic effect on these MCs in a dose-dependent manner and significantly induced caspase activities (Figure 4e and f). Compared with the TCs, the resistance of MCs to BKM120 only ranged from 4.61- to 5.73-folds (Table 1), which indicated that BKM120 could kill MCs more efficiently than DOX and etoposide.

**Figure 5:**
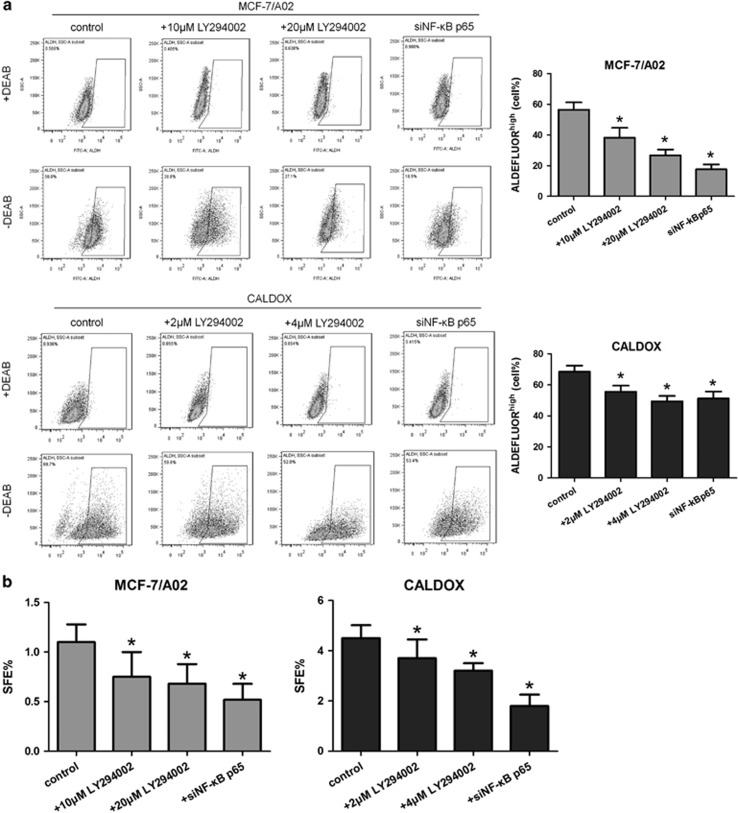
Inhibiting the PI3K/AKT/NF-*κ*B signaling pathway diminishes breast cancer stem cell population. (**a**) Flow cytometry analysis of ALDH activity. Cells with LY294002 treatment for 48 h or after siRNA transfection for 72 h were assayed with an Aldefluor assay kit in the presence and absence of ALDH inhibitor DEAB. Gating in the control was set up to a maximum of 1% of cells. Representative plots of three independent experiments are shown. Quantitative data show the average percentage of ALDH^high^ cells±S.D. of three independent experiments (right panel). (**b**) Mammosphere-forming efficacy of cells with LY294002 treatment or siRNA transfection. SFE was calculated as the number of spheres formed in 10 days divided by the original number of single cells seeded and expressed as a percentage. Bars represent the mean percentage of mammospheres±S.D. from three separate experiments. *P<0.05

To ascertain that blockage of the PI3K/AKT/NF-*κ*B cascade was functionally important for BKM120-induced stemness reduction in MDR cell lines, we used LY294002 and NF-*κ*B p65 siRNA to specifically inhibit PI3K/AKT activity and NF-*κ*B p65 expression. Figure 5 confirmed that abolishing PI3K/AKT signaling or NF-*κ*B p65 silencing was sufficient to decrease ALDH^high^ population as well as SFE in MCF-7/A02 and CALDOX cells.

### BKM120 is synergistic with chemotherapeutic agents in MDR breast cancer cells

Alterations in the PI3K/AKT signaling pathway have been shown to track consistently with therapy-induced resistance in breast cancer patients, including endocrine-based therapy, chemotherapy and HER2-targeted therapy.^36, 37, 38^ Recent studies demonstrate that targeting the PI3K/AKT pathway in combination with trastuzumab or tamoxifen is beneficial in trastuzumab-resistant breast cancer or endocrine therapy-resistant breast cancer.^38, 39^ Hence, we further investigated whether BKM120 could synergize with chemotherapeutic agents commonly used in breast cancer therapy regimens. For this purpose, MCF-7/A02 and CALDOX cells were treated with increasing concentrations of DOX/etoposide, either alone or in combination with BKM120 at fixed ratios (DOX/BKM120, 5:1 for MCF-7/A02; etoposide/BKM120, 15:1 for MCF-7/A02; DOX/BKM120, 1.25:1 for CALDOX; and etoposide/BKM120, 25:1 for CALDOX). As shown in Table 2, the combination index (CI) values ranged from 0.1 to 0.6, indicating that DOX or etoposide and BKM120 used in combination acted synergistically in MCF-7/A02 and CALDOX cells.

As P-gp (encoded by *mdr-1*) overexpression is the most crucial mechanism that contributes to MDR phenotype in many cancer cells including MCF-7/A02, we examined whether BKM120 modulates P-gp expression. RT-qPCR results showed that, although administration of 8 *μ*M BKM120 caused almost 50% of the cells undergoing apoptosis, the mRNA levels of *mdr-1* in MCF-7/A02 remained constantly elevated (Supplementary Figure S6A). Furthermore, the Rhodamine 123 retention in the cells as detected with flow cytometry demonstrated that intracellular Rhodamine 123 levels were not enhanced in MCF-7/A02 cells after BKM120 treatment (Supplementary Figure S6C). The MDR phenotype of CALDOX did not involve drug transporters, as resistant cell-accumulated Rhodamine 123 was comparable to the parental cells (Supplementary Figure S6C). It has been recently reported that chemoresistance of CALDOX is partially caused by the downregulation of TOP2A.^28^ In accordance with the previous finding, RT-qPCR analysis showed that TOP2A mRNA levels were significantly lower in CALDOX cells than their parental cells. However, BKM120 did not alter TOP2A expression (Supplementary Figure S6B). These findings suggest that the increase in MDR breast cancer cell sensitivity to chemotherapeutic agents by BKM120 is independent of P-gp and TOP2A expression.

### Effect of BKM120 on xenograft tumor growth of MCF-7/A02 and CALDOX cells in nude mice

**Figure 6:**
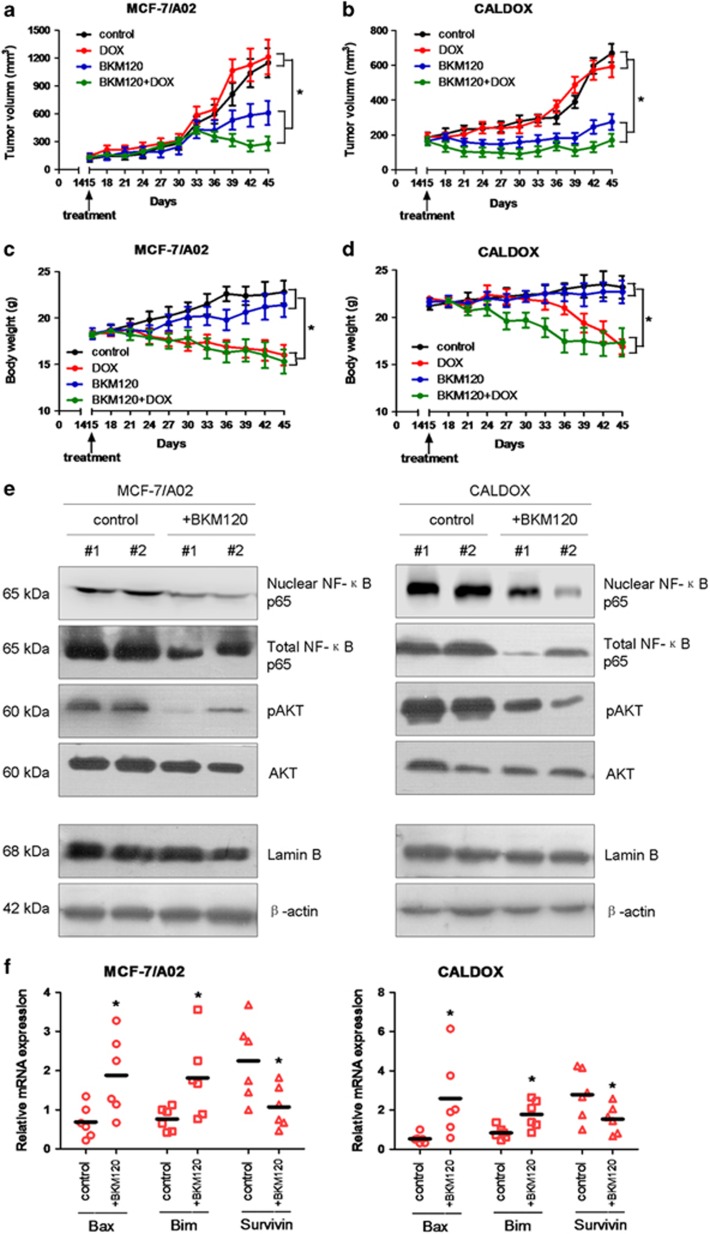
The antitumor activity of BKM120 in MCF-7/A02 and CALDOX xenograft tumors. (**a** and **b**) Tumor sizes of MCF-7/A02 (**a**) and CALDOX (**b**) xenografts after treatment with PBS (control), DOX, BKM120 or BKM120 plus DOX. Data are presented as the mean tumor size±S.D. of six mice per group. (**c** and **d**) Body weight of nude mice bearing MCF-7/A02 (**c**) and CALDOX (**d**) xenografts treated with PBS (control), DOX, BKM120 or BKM120 plus DOX. Data are presented as the mean body weight±S.D. of six mice per group. (**e**) Western blot analysis of pAKT, AKT, nuclear NF-*κ*B p65 and total NF-*κ*B p65 on MCF-7/A02 and CALDOX derived tumors treated with BKM120 or PBS. Tumors were obtained from two mice randomly chosen from six mice per group. Both lamin B and *β*-actin were used as loading controls. (**f**) Relative fold of Bax, Bim and Survivin gene expression levels in MCF-7/A02 and CALDOX derived tumors treated with BKM120 or PBS. *P<0.05

The significant antitumor activity of BKM120 on chemoresistant breast cancer cells *in vitro* led us to investigate whether its antitumor efficacy would be maintained *in vivo*. MCF-7/A02 and CALDOX cells were injected into the mammary fat pad of female nude mice. On day 14 after injection, mice were randomly divided into four groups with equal number of mice. Each group was treated with BKM120, DOX, BKM120 plus DOX or vehicle control, respectively. As expected, the tumor continued to grow in DOX-treated groups in both xenograft models indicating DOX resistance, whereas BKM120 alone substantially curtailed tumor progression in both xenograft models relative to vehicle control and DOX-treated groups (Figure 6a and b). Moreover, the animals receiving both DOX and BKM120 exhibited an even more dramatic reduction of tumor growth in either MDR cell xenograft (Figure 6a and b). Interestingly, the animals receiving DOX alone displayed a significant body weight (BW) loss (~30% reduction by day 45 as compared with the BW on day 15). In contrast, no significant BW loss was observed in the animals treated with BKM120 alone in either MDR cell xenograft (Figure 6c and d).

Consistent with our *in vitro* results, western blot results revealed that BKM120 treatment reduced phospho-AKT, total and nuclear NF-*κ*B p65 subunit protein levels in xenograft tumor tissues (Figure 6e). Apoptosis-related genes including *Bax*, *Bim* and *Survivin* were also regulated by BKM120 *in vivo* (Figure 6f). Thus, BKM120 effectively blocks the aberrant activity of the PI3K/AKT/NF-*κ*B signaling pathway in chemoresistant breast cancer cells, which subsequently induces xenograft tumor regression *in vivo*.

## Discussion

Most cytotoxic anticancer therapies are encumbered by the development of acquired resistance of cancer cells. Chemoresistance is a complex phenomenon involving multiple mechanisms. Reduction of drug accumulation, enhancement of DNA repair, impediment to apoptosis and alterations in cell cycle are believed to be the major causes of chemoresistance. Many of these factors are manifested in intracellular signaling pathways, and one of the most prominent is the PI3K/AKT pathway.

Here, we employed three drug-resistant human breast cancer cell lines for the experiments, MCF-7/A02, CALDOX and MD60, all of which possess the MDR phenotype but are caused by different mechanisms. The overexpression of P-gp has been recognized as the most significant factor conferring chemoresistance of MCF-7/A02 and MD60. In contrast, compared with Cal51, CALDOX cells have no increased P-gp expression or any other drug transporters, implying that mechanisms independent of drug transporters have a leading role in chemoresistance of CALDOX cells. Despite that the causes of drug resistance in these cell lines are different, the PI3K/AKT/NF-*κ*B signaling is overactivated in all three cell lines, which is consistent with the findings reported by other groups.^10, 12^ Indeed, this crucial survival pathway promotes acquired resistance in a wide range of cancers, such as breast cancer, leukemia, ovarian cancer and non-small-cell lung carcinoma.^39, 40, 41^ Hence, novel therapeutics against this PI3K/AKT/NF-*κ*B pathway may offer new strategies to overcome drug resistance.

In this study, we examined the responses of three chemoresistant breast cancer cell lines to the PI3K inhibitor BKM120. Our studies were performed both *in vitro* and *in vivo*. The inhibition of PI3K by BKM120 results in a dramatic decrease in AKT phosphorylation and subsequent downregulation of NF-*κ*B. Furthermore, activation of caspase-3,7,9, inhibition of survivin expression, as well as augment of Bax and Bim expression, were also detected after BKM120 treatment. These are consistent with recent studies that BKM120 elevated Bim expression in chronic lymphocytic leukemia cells^42^ and induced Bax expression and caspase-3/7 activation in glioma cell lines, T-cell acute lymphoblastic leukemia and ER-positive breast cancer cells.^19, 43, 44^ It is well known that AKT is a central node in the PI3K signaling pathway that activates a number of downstream pathways implicated in tumorigenesis. BKM120 has previously been shown to inhibit some other pathways, including the AKT/mTOR pathway in breast cancer and hepatocellular carcinoma, the AKT/GSK3/FBXW7 pathway in chronic lymphocytic leukemia cells and the AKT/FOXO3a axis in lung cancer cells.^42, 45, 46, 47^ Our study uncovers for the first time that BKM120 can also modulate NF-*κ*B, another critical substrate of AKT. Importantly, NF-*κ*B signaling and cell apoptosis are frequently associated with many solid cancers,^48, 49^ although the molecular mechanisms remain obscure. It is tempting to speculate that AKT/NF-*κ*B represents a ‘salvage pathway' that links BKM120 with apoptosis induction.

Comparing traditional chemotherapeutic agents such as doxorubicin and etoposide, BKM120 reduced cell viability more effectively in a concentration-dependent manner in all tested MDR breast cancer cells. Others also proved that therapy resistance could be partially overcome by downregulating AKT/NF-*κ*B. For instance, salinomycin induced apoptosis in cisplatin-resistant ovarian cancer cells through inhibiting the AKT/NF-*κ*B pathway.^50^ PS1145 can overcome imatinib resistance in leukemia through blocking the NF-*κ*B pathway.^29^ LY294002, another important selective inhibitor of PI3K, has been proven to overcome acquired resistance of 5-FU in gastric cancer by modulating NF-*κ*B activity.^51^ Our study also demonstrated that the AKT/NF-*κ*B pathway blocked by LY294002 or NF-*κ*B p65 siRNA led to apoptosis of MDR breast cancer cells. Although some studies reported that several AKT/NF-*κ*B inhibitors reverse the MDR phenotype by decreasing P-gp expression,^52, 53^ our data indicated that BKM120 did not alter P-gp expression, suggesting that BKM120 overcame chemoresistance through inducing apoptosis, rather than increasing drug influx.

Accumulating evidence indicates that breast CSCs have a crucial role in therapy resistance and recurrence of breast cancers.^5, 54^ Hence, breast CSCs are considered to be critical therapeutic targets, and elimination of breast CSCs may improve the outcomes of cancer chemotherapy.^55^ Recent studies indicate that the activation of the PI3K/AKT/NF-*κ*B pathway is indispensable for maintaining the stemness and chemoresistance of breast CSCs.^56, 57^ Present and other studies have shown that PI3K inhibition sensitizes CSCs to chemotherapy and molecular targeted therapy in several cancers including leukemia,^58^ hepatocellular carcinoma^59^ and breast cancer.^60^ Moreover, the blockage of NF-*κ*B activity also provokes cytotoxic effects on CSCs in glioblastoma multiforme.^61^ In line with these results, our findings revealed that, in contrast to highly resistant to traditional chemotherapeutic agents, the CSC population of the three MDR cell lines tested remained relatively sensitive to BKM120-induced cytotoxicity. Furthermore, BKM120 remarkably eliminated CSCs in drug-resistant cells. After exposure to BKM120, the clonogenicity of resistant cells was evidently eradicated *in vitro*, and the ALDH^high^ and CD44^high^/CD24^low^ population decreased obviously in the survival cells. In addition, LY294002 and NF-*κ*B silencing also inhibited stemness significantly, indicating that targeting PI3K/AKT/NF-*κ*B pathway is emerged as a promising approach to kill CSCs and consequently surmount MDR in breast cancers.

BKM120 exerted a synergistic effect with doxorubicin both *in vitro* and *in vivo*. It has recently been reported that BKM120 in combination with trastuzumab is beneficial in trastuzumab-resistant breast cancer.^62^ Other PI3K inhibitors, including LY294002 and buparlisib, have also been proven to have greater clinical efficacy combined with endocrine therapy.^63, 64^ In some other solid tumors, BKM120 has been reported to synergistically work with an mTOR inhibitor RAD001 and a Bcl-2 inhibitor ABT-737 in suppression of lung cancer^18^ and glioblastoma cell growth,^19^ respectively. It is of note that these combined therapies were all well tolerated. Consistently, our study demonstrates that BKM120 sensitizes MDR breast cancer cells to cytotoxic drugs (doxorubicin and etoposide) using CI-isobologram analysis, suggesting that combination with BKM120 therapy can augment the cytotoxic activity of chemodrugs against MDR breast cancer growth but with no further toxicities.

Overall, the present study discloses that the MDR phenotype of breast cancer cells is associated with an aberrant activation of the PI3K/AKT/NF-*κ*B signaling pathway. Our findings establish that BKM120 effectively inhibits this signaling pathway, and provide strong evidence demonstrating that BKM120 potently induces cell apoptosis and aggressively eliminates breast CSCs in these MDR breast cancer cells. Moreover, the combination therapy of BKM120 and doxorubicin shows a synergistic effect both *in vitro* and *in vivo*. Our data suggest that targeting the PI3K/AKT/NF-*κ*B signaling pathway using selective inhibitors such as BKM120 can be a potential strategy for treatment of relapsed MDR breast cancers.

## Materials and Methods

### Cell culture

Human breast cancer cell line MCF-7 and its MDR counterpart MCF-7/A02 were gifts from Professor Dongsheng Xiong (Institute of Hematology, PUMC, Tianjin, China) and were cultured as previously described.^65^ Human breast cancer cell line Cal51 and its MDR counterpart CALDOX were gifts from Dr. Ernesto Yague (Imperial College London, UK) and were cultured as previously described.^28^ MTMEC and its doxorubicin-resistant derivative MD60 were also gifts from Dr. Ernesto Yague, and they were routinely maintained in a serum-free HuMEC medium (Life Technologies, Paisley, UK) as previously described.^66^ MTMEC is an immortalized human mammary epithelial cell line expressing TERT, SV40 large T antigen, a constitutively active form of PI3K, p110*α* and oncogenic RAS.^67^ These cells were treated with chemotherapeutic drugs doxorubicin, etoposide, taxol (Sigma, St. Louis, MO, USA), PI3K inhibitors LY294002 (Cell Signaling Technology, Danvers, MA, USA) and NVP-BKM120 (Novartis, Basel, Switzerland) alone or in combination at various doses and durations.

### Cell viability analysis

MTT (3-(4,5-Dimethylthiazol-2-yl)-2,5-diphenyltetrazolium bromide) assays were performed to evaluate the cell viability in response to drug treatments and were also used to determine the concentration of drug that inhibited cell growth by 50% (IC_50_) after 3 days of treatment.^68^ For drug combination experiments, a CI number was calculated using the CalcuSyn software (Biosoft, Cambridge, UK) based on the Chou and Talalay method. CI values between 0.1 and 0.9 define different grades of synergism: values between 0.9 and 1.1 are additive, whereas values >1.1 are antagonistic.

### Drug resistance clonogenic assay

Cells at a density of 2 × 10^5^ cells/well in six-well plates were treated with a single dose of doxorubicin (3 *μ*M for MCF-7 and MCF-7/A02, 0.2 *μ*M for Cal51 and CALDOX), or 2 *μ*M BKM120 for 1 week. Resistant clones were fixed with 4% paraformaldehyde and stained with 0.2% crystal violet and counted. Crystal violet retained in the cells was solubilized with 0.5% acetic acid and quantified by measurement of optical density at 592 nm.

### siRNA transfection

Cells at a density of 3 × 10^5^ cells/well in six-well plates were used for siRNA transfection. Briefly, 50 nM siRNA against the NF-*κ*B p65 subunit or scrambled siRNA control (GenePharma Company, Shanghai, China) was mixed with lipofectamine 3000 (Life Technologies, Grand Island, NY, USA) and then added to each well. The effects of siRNA on NF-*κ*B p65 subunit mRNA and protein levels were examined 48 and 72 h after transfection, respectively.

### Protein extraction and western blotting

A modified RIPA buffer (50 mM Tris–HCl, 150 mM NaCl, 0.25% SDS, 1% Triton X-100, 0.25% sodium deoxycholate, 1 mM EDTA, 1 mM EGTA and 1 mM dithiothreitol) with protease inhibitor cocktail (Sigma) was used for protein isolation from whole cells or nuclear fraction. Cell nuclei were isolated using a Nuclei EZ Prep Kit (Sigma) according to the instruction recommended by the manufacturer. Protein concentrations were determined using the BCA Protein Assay Kit (Pierce, Rockford, IL, USA). An equal quantity (50 *μ*g) of proteins was resolved on 12% polyacrylamide gels and transferred to nitrocellulose membranes (Millipore, Billerica, MA, USA), and then blocked with 5% blotting grade milk (Bio-Rad, Hercules, CA, USA) in PBST (0.1% Tween 20 in phosphate-buffered saline (PBS)). The membranes were incubated with primary antibodies to phospho-AKT (D9E), AKT1 (C73H10), Bax (D2E11), Bim (C34C5; Cell Signaling Technology), Survivin (ab76424, Abcam, Cambridge, UK) and NF-*κ*B p65 (sc372, Santa Cruz Biotechnology, Dallas, TX, USA) at 4 °C overnight. The peroxidase-conjugated anti-rabbit IgG secondary antibody (Cell Signaling Technology) was incubated with the membranes for 2 h at room temperature. Immunoblotting signals were detected using the SuperSignal West Pico Chemiluminescent Substrate (Pierce) according to the instruction suggested by the manufacturer. All the membranes were re-probed with anti-*β*-actin (sc47778) or lamin B (sc6216) antibodies (Santa Cruz Biotechnology), which served as loading controls.

### RNA isolation and RT-qPCR

Total RNA was isolated using Trizol (Invitrogen, Carlsbad, CA, USA) following the procedure suggested by the manufacturer. The cDNA was generated using OligdT primers and SuperScript III reverse transcriptase (Invitrogen) using 2 *μ*g total RNA. Specific primers for each gene (Supplementary Table S3) were designed using the Primer Express program (Applied Biosystems, Foster City, CA, USA). Quantitative real-time PCR was carried out using SYBR Green I (Takara, Dalian, China) and detected using an ABI SDS7900 Real-time PCR system (Applied Biosystems). A standard curve for each gene was included in each PCR amplification for calculation of the *C*_t_ value. Relative transcript levels were normalized with RPS14.

### Annexin V staining

Cell apoptosis was assessed using an Annexin V- fluorescein isothiocyanate (FITC) and PI double-staining apoptosis detection kit (Becton Dickinson, Mountain View, CA, USA) according to the procedure suggested by the manufacturer. Briefly, cells (2 × 10^5^) were washed twice with PBS and suspended in 100 *μ*l binding buffer followed by staining with 5 *μ*l Annexin V-FITC and 5 *μ*l PI for 15 min in the dark at room temperature. The fluorescence was measured with a flow cytometer (BD FACSCanto II, Becton Dickinson). Quantitative data show the average percentage of annexin V-positive cells (both in early apoptosis, LR quadrant, and late apoptosis, UR quadrant) of three independent experiments.

### Drug accumulation assay

The relative cellular accumulation of tracer dye Rhodamine123 (Sigma) was determined using flow cytometry as described before.^69^ Briefly, MCF-7/A02 cells were collected 48 h after being treated with BKM120 and then incubated with 0.3 *μ*M Rhodamine123 in RPMI1640 medium with constant shaking for 90 min at 37 °C. After washing twice with ice-cold PBS, the cells were resuspended in 500 *μ*l ice-cold PBS. Rhodamine123 accumulation in cells was determined using a flow cytometer (BD FACSCanto II, Becton Dickinson). The cells were excited at 488 nm, and emission was measured at 530 nm.

### Analysis of CD44^+^/CD24^–^ cell subpopulation

For stem cell marker analysis, APC-conjugated CD44 and phycoerythrin (PE)-conjugated CD24 monoclonal antibodies (BD Biosciences, San Jose, CA, USA) were used as described by Lombardo *et al.*^31^ Briefly, cells were harvested 48 h after treatment and suspended in PBS at 1 × 10^6^ cells/ml. Both APC-CD44 and PE-CD24 antibodies or their respective isotype controls APC-IgG and PE-IgG were added to the cell suspensions at concentrations recommended by the manufacturer and incubated for 40 min at 4 °C in the dark. The labeled cells were washed in PBS and then analyzed with a flow cytometry (BD FACSCanto II, Becton Dickinson). Gating was set to relevant isotype control (APC-IgG and PE-IgG)-labeled cells for each cell line.

### Aldefluor assay

An Aldefluor assay kit (StemCell Technologies, Vancouver, BC, Canada) was used for the determination of ALDH activity with flow cytometry as described by Hu *et al.*^66^ Briefly, cells were harvested 48 h after treatment and suspended in Aldefluor assay buffer at 1 × 10^6^ cells/ml. As a negative control, half the sample was transferred to a tube containing 5 *μ*l of the ALDH inhibitor diethylaminobenzaldehyde. Activated Aldefluor substrate (5 *μ*l) was added to both samples and incubated at 37 °C for 45 min to allow substrate conversion. The cells were resuspended in Aldefluor assay buffer and analyzed using a flow cytometer (BD FACSCanto II, Becton Dickinson).

### Electrophoretic mobility shift assay

Electrophoretic mobility shift assay (EMSA) for NF-*κ*B was performed using Lightshift Chemiluminescent EMSA kits (Thermo Fisher Scientific, Bonn, Germany) according to the procedure suggested by the manufacturer. Annealed oligonucleotides containing NF-*κ*B-binding site (5′-AGTTGAGGGGACTTTCCCAGGC-3′ and 3′-TCAACTCCCCTGAAAGGGTCCG-5′) were end-labeled using a biotin 3′end-labeling kit (Thermo Fisher Scientific). Briefly, 5 pmol double-stranded oligonucleotides were incubated in a 50*-μ*l reaction buffer containing 10 *μ*l of 5 × terminal deoxynucleotidyl transferase (TdT) buffer, 5 *μ*l of 5 *μ*M biotin-N-4-CTP, 10 U of diluted TdT and 25 *μ*l of ultra-pure water at 37 °C for 30 min. DNA–protein complexes were prepared by mixing 50 fmol biotin-end-labeled oligonucleotides and 5 *μ*l nuclear extracts in 1 × binding buffer. DNA–protein complexes and free DNA were separated on a native polyacrylamide gel and transferred to a nylon membrane. The membrane was subsequently incubated with streptavidin–horseradish peroxidase conjugate and a chemiluminescent substrate. DNA–protein complexes were visualized by exposure to X-ray films.

### Caspase activity measurement

Cells (1 × 10^4^) were incubated with BKM120 or LY294002 in a 96-well plate for 24 h. The caspase-3/7 and caspase-9 activities were measured using the Caspase-Glo 3/7 Assay and Caspase-Glo 9 Assay Kits, respectively (Promega, Madison, WI, USA) following the protocol recommended by the manufacturer. Fluorescence was measured using a GloMax 20/20 Luminometer (Promega). z-VAD-fmk (Selleck, Houston, TX, USA) was used as a pancaspase inhibitor.

### Immunofluorescence staining

Cells were seeded on square coverslips in six-well plates. Subsequently, cells were fixed with 2% paraformaldehyde for 15 min, permeabilized with 0.2% Triton X-100 for 5 min and blocked with 3% BSA for 30 min. The coverslips were then incubated with rabbit anti-NF-*κ*B p65 primary antibody for 1 h at room temperature followed by Alexafluor 488-labeled secondary antibody (Santa Cruz Biotechnology) for 1 h at room temperature. The coverslips were counterstained with 1 mg/ml 4′,6-Diamidino-2-phenylindole (Sigma) for 10 min to visualize the cell nucleus. All antibodies and staining reagents were diluted in PBS containing 3% BSA. The cells were washed three times with PBS after each step of the staining procedures. The coverslips were covered with ProLong Gold antifade mounting medium (Invitrogen) and then were viewed and photographed with a Zeiss LSM 510 Meta fluorescence microscope (Zeiss, Jena, Germany).

### Soft agar colony formation assay

Cells were disassociated and suspended in DMEM or HuMEC medium containing 0.3% agar and plated on the top of a solidified layer of 0.6% agar. The cells were plated at a density of 2 × 10^5^ cells/well in six-well plates, and the colonies were counted 21 days later.

### Mammosphere culture

Cells (1 × 10^3^) were plated in each well of an ultralow attachment plate (Corning Incorporated, Corning, NY, USA) with 3 ml serum-free mammary epithelial growth medium (MEGM, BioWhittaker, Walkersville, MD, USA), supplemented with B27 (Invitrogen), 20 ng/ml EGF and 20 ng/ml basic FGF (BD Biosciences). Colony formation was assessed 10 days later.

### *In vivo* xenografts

Cells (1 × 10^7^) were suspended in 100 *μ*l PBS containing 50% Matrigel (BD Biosciences) and injected into the mammary fat pad of 4–5-week-old female nude mice (Vital River Company, Beijing, China). Tumor sizes were measured every 3 days in two dimensions using a caliper, and the tumor volume was calculated with the following formula: tumor volume (mm^3^)=0.5 × *ab*^2^ (*a* and *b* being the longest and shortest diameters of the tumor, respectively). Fourteen days after cell injection, the tumor-bearing mice were randomly divided into four groups (six animals/group): (1) control group (normal saline), (2) BKM120 group (50 mg BKM120 per kg BW), (3) DOX group (2 mg doxorubicin per kg BW) and (4) BKM120+DOX group (50 mg BKM120 and 2 mg DOX per kg BW). Drugs were injected every 3 days, and tumor volumes were monitored till the mice were killed. Mice were killed in a humane manner, and the tumors were collected for protein extraction and RNA extraction. All mice were maintained as required under the National Institutes of Health guidelines for the Care and Use of Laboratory Animals. The use of animals in this study has been approved by the Animal Care and Use Committee of Tianjin Cancer Hospital.

### Statistical analysis

Comparisons of the means among more than two groups were performed by one-way analysis of variance. Student's *t*-test was used when comparing the means of two groups. A *P*-value<0.05 was considered statistically significant.

## Figures and Tables

**Table 1:** Mammosphere cells of MCF-7/A02 and CALDOX sensitivity to different drugs

**Drug** (*μ*M)	**IC**_**50**_ **of MCF-7/A02**		**Mammosphere-cell resistance ratio**
	**Total cells**	**Mammosphere cells**	
Doxorubicin	128.3	8827	68.80
Etoposide	206.8	8747	42.30
BKM120	5.6	32.1	5.73
**Drug** (*μ*M)	**IC**_**50**_ **of CALDOX**		**Mammosphere-cell resistance ratio**
	**Total cells**	**Mammosphere cells**	
Doxorubicin	5.73	272.7	47.59
Etoposide	26.8	487.8	18.20
BKM120	1.8	8.3	4.61

**Table 2:** Cytotoxicity of BKM120 and doxorubicin/etoposide to different breast cancer cell lines

*MCF-7/A02*					
BKM120 (*μ*M)	1	2	4	8	16
Doxorubicin (*μ*M)	5	10	20	40	80
CI	0.368	0.279	0.184	0.241	0.144
BKM120 (*μ*M)	1	2	4	8	16
Etoposide (*μ*M)	15	30	60	120	240
CI	0.262	0.531	0.355	0.471	0.138
					
*CALDOX*					
BKM120 (*μ*M)	0.5	1	2	4	8
Doxorubicin (*μ*M)	0.625	1.25	2.5	5	10
CI	0.306	0.213	0.362	0.212	0.129
BKM120 (*μ*M)	0.5	1	2	4	8
Etoposide (*μ*M)	12.5	25	50	100	200
CI	0.351	0.412	0.501	0.522	0.223

## Abbreviations

ALDH: aldehyde dehydrogenase
BKM120: NVP-BKM120
DOX: doxorubicin
MDR: multidrug resistance
NF-*κ*B: Nuclear factor kappa B
P-gp: P-glycoprotein
PI3K: phosphatidylinositol 3-kinase
SC: stem-like cells
