# Supplementary material for: Effects of PI3K inhibitor NVP-BKM120 on overcoming drug resistance and eliminating cancer stem cells in human breast cancer cells
Source: Cell Death Dis. 2015 Dec 17;6(12):e2020–. doi: 10.1038/cddis.2015.363 (PMC4720896; doi:10.1038/cddis.2015.363)
Supplement: Supplementary Information [file cddis2015363x1.doc]

**Supplementary Information**

**Effects of PI3K inhibitor NVP-BKM120 on overcoming drug resistance and eliminating cancer stem cells in human breast cancer cells**

*Yunhui Hu^1,2 *†^, Rong Guo^1 *^, Jiayang Wei^1^, Yang Zhou^1^,Wei Ji^2^, Jingjing Liu^1^, Xiangcheng Zhi^1^ and Jin Zhang^1,2 †^*

*^1^* *The 3rd Department of Breast Cancer, China Tianjin Breast Cancer Prevention, Treatment and Research center,* *Tianjin Medical University Cancer Institute and Hospital, National Clinical Research Center of Cancer,* *Huan Hu Xi road, Ti Yuan Bei, He xi district, Tianjin, 300060, PR China*

*^2^ Key laboratory of breast cancer prevention and therapy of ministry of education, Huan Hu Xi road, Ti Yuan Bei, He xi district, Tianjin, 300060, PR China*

*^*^ These authors contributed equally to this work.*

*^†^ Senior corresponding authors contributed equally*

*Jin Zhang: The 3rd Department of Breast Cancer, Tianjin Medical University Cancer Institute and Hospital, Huan-Hu-Xi road,Ti-Yuan-Bei,He xi district, Tianjin, 300060, PR China. Tel: +86-22-23340123; Email:* [*zhangjin@tjmuch.com*](mailto:zhangjin@tjmuch.com)

*Yunhui Hu: The 3rd Department of Breast Cancer, Tianjin Medical University Cancer Institute and Hospital, Huan-Hu-Xi road,Ti-Yuan-Bei,He xi district, Tianjin, 300060, PR China. Tel: +86-13702046550; Email:* [*yunhuihu200408@163.com*](mailto:yunhuihu200408@163.com)

**
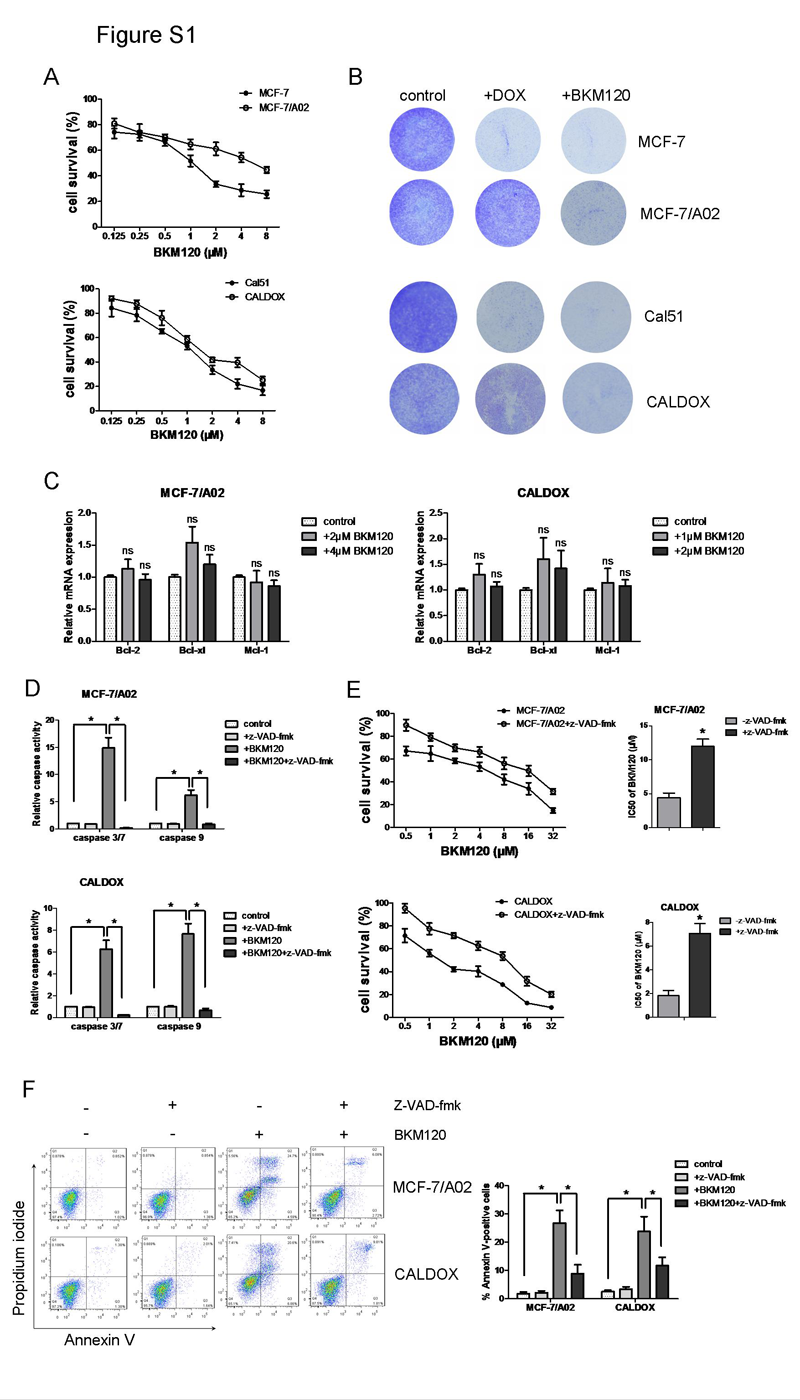
**

**Fig. S1.** **BKM120 reduced cell viability in both of chemosensitive and chemoresistant breast cancer cells. (A)** Dose-response curves used to calculate the IC_50_ of BKM120 for MCF-7 and MCF-7/A02 (upper), as well as Cal51 and CALDOX (lower). **(B)** Cells were treated with doxorubicin (DOX, 3 μM for MCF-7 and MCF-7/A02, 0.2 μM for Cal51 and CALDOX) and BKM120 (2 μM for all cell lines) for seven days and the cells were stained with crystal violet. Pictorial data are representative pictures of three independent replicates. **(C)** Bcl-2, Bcl-xl and Mcl-1 mRNA levels in MCF-7/A02 and CALDOX cells determined by RT-qPCR after BKM120 treatment at various concentrations for 48 h. **(D)** Caspase-3/7 and Caspase-9 activities of MCF-7/A02 (upper panel) and CALDOX (lower panel) after treatments with 4 μM BKM120 and/or 2 μM z-VAD-fmk. **(E)** Dose-response curves and IC_50_ values of BKM120 for MCF-7/A02 (upper panel) and CALDOX (lower panel) pretreated with 2 μM z-VAD-fmk for 4 hours or untreated. **(F)** Cells were treated with 2 μM BKM120 and/or 2 μM z-VAD-fmk. Annexin V/PI staining was detected by flow cytometry. Representative plots of three independent experiments are shown. Quantitative data show the average percentage of annexin V positive cells (both in early apoptosis, lower right quadrant, and late apoptosis, upper right quadrant) of three independent experiments (right panel). Data are presented as mean±SD of three independent MTT assays.


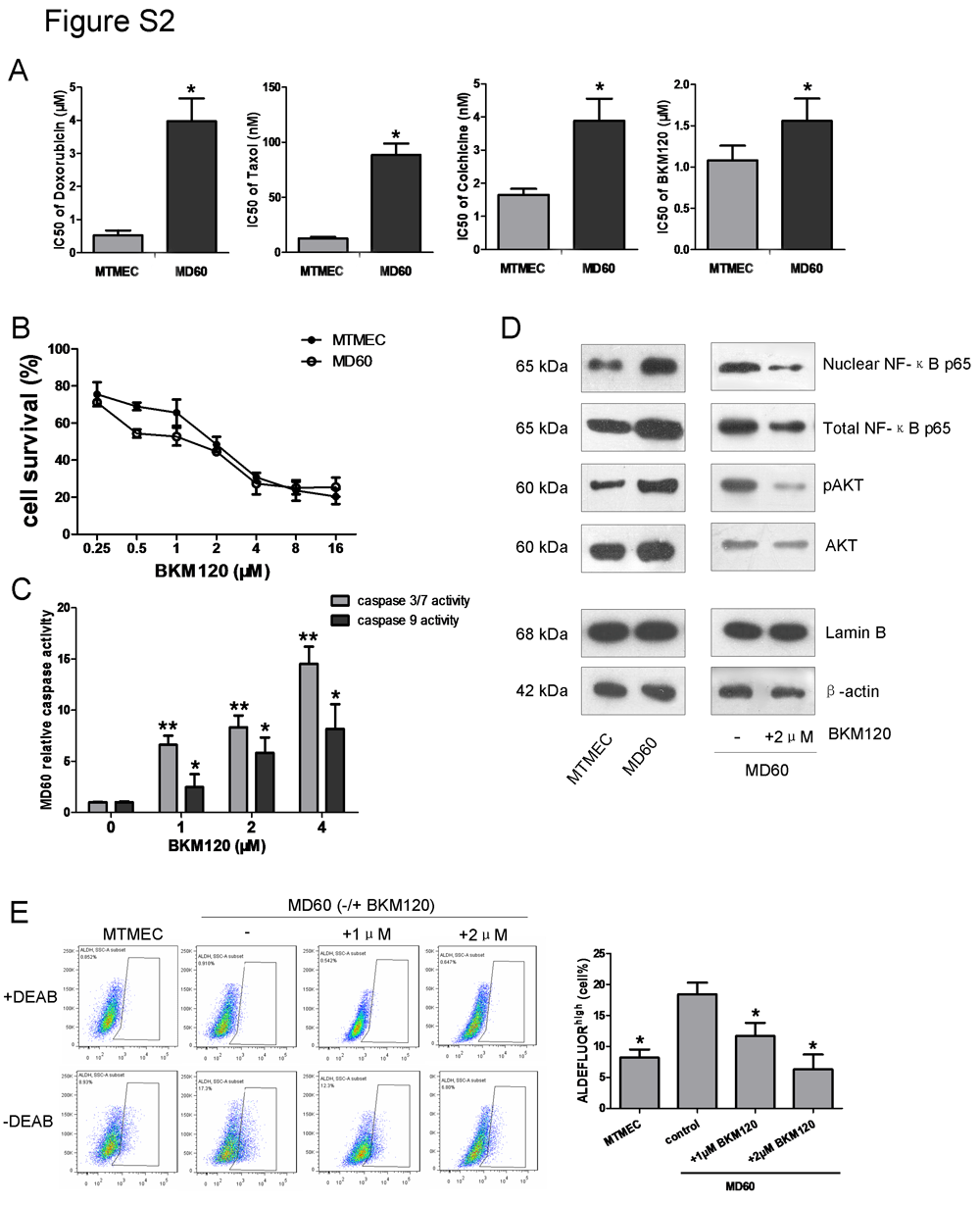


**Fig. S2. Efficacy of BKM120 in MTMEC and MD60 cell lines. (A)** IC_50_ values of different drugs were measured in MTMEC and MD60 cells. **(B)** Dose-response curves used to calculate the IC_50_ of BKM120 for MTMEC and MD60 cells. Data are presented as mean ± SD of three independent MTT assays. **(C)** Caspase-3/7 and Caspase-9 activities of MD60 cells after treatments with various BKM120 concentrations. **(D)**Western blots show the protein levels of phospho-AKT (pAKT), AKT, nuclear NF-κB p65 and total NF-κB p65 in MTMEC, MD60 and MD60 cells after BKM120 treatment. β-actin was used as a loading control for pAKT, AKT and total NF-κB p65. Lamin B was used as a loading control for nuclear NF-κB p65. **(E)** Cells with or without BKM120 treatment for 48 h were assayed with an Alderfluor assay kit in the presence and absence of the ALDH inhibitor DEAB. Representative plots of at least three independent experiments are shown. Quantitative data show the average percentage of ALDH^high^ cells ± SD of three independent experiments (right panel).

**
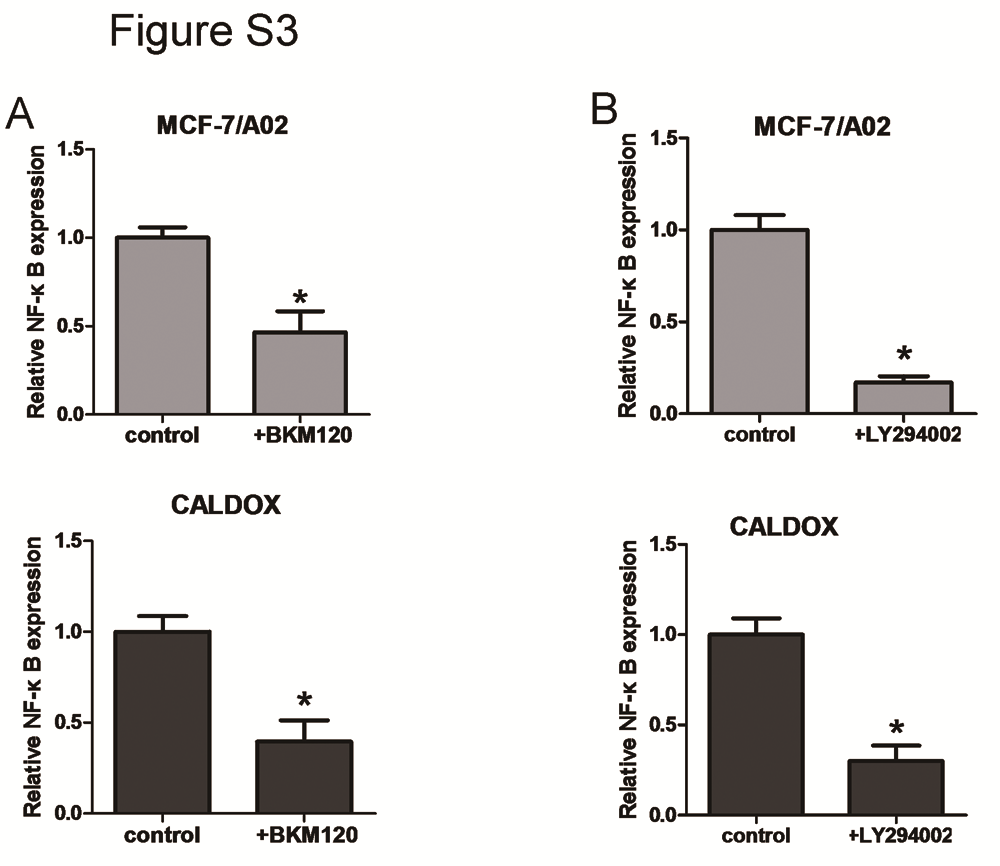
**

**Fig. S3. PI3K inhibitors suppress NF-κB p65 expression on mRNA level (A)** Fold changes of NF-κB p65 mRNA levels determined by RT-qPCR in MDR cells after BKM120 treatments (4 μM for MCF-7/A02 and 2 μM for CALDOX) for 48 h. **(B)** Fold changes of NF-κB p65 mRNA levels determined by RT-qPCR in MDR cells after LY294002 treatments (40 μM for MCF-7/A02 and 4 μM for CALDOX) for 48 h.


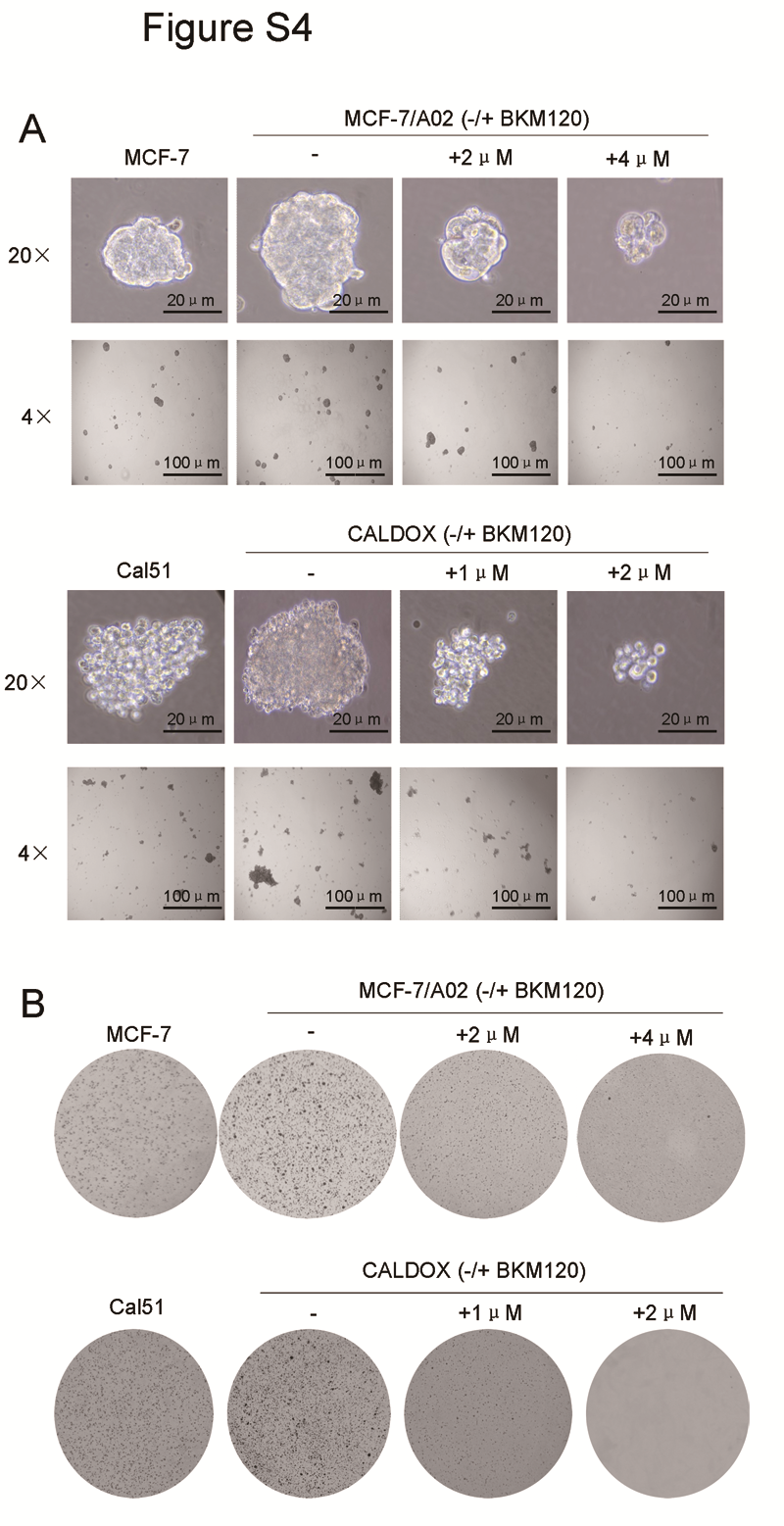


**Fig. S4. BKM120 reduces the stem cell sub-population of MCF-7/A02 and CALDOX cells.** **(A)** Mammosphere formation in MDR and their parental cells. Representative primary mammosphere pictures are shown for each cell line. Magnification 20×for upper panel and 4×for lower panel. **(B)** Soft agar colony formation assay was performed by plating MDR and their parental cells in soft agar and treated with or without BKM120. Day of plating was designated as day 0. Images were taken at day 21 after staining with MTT.


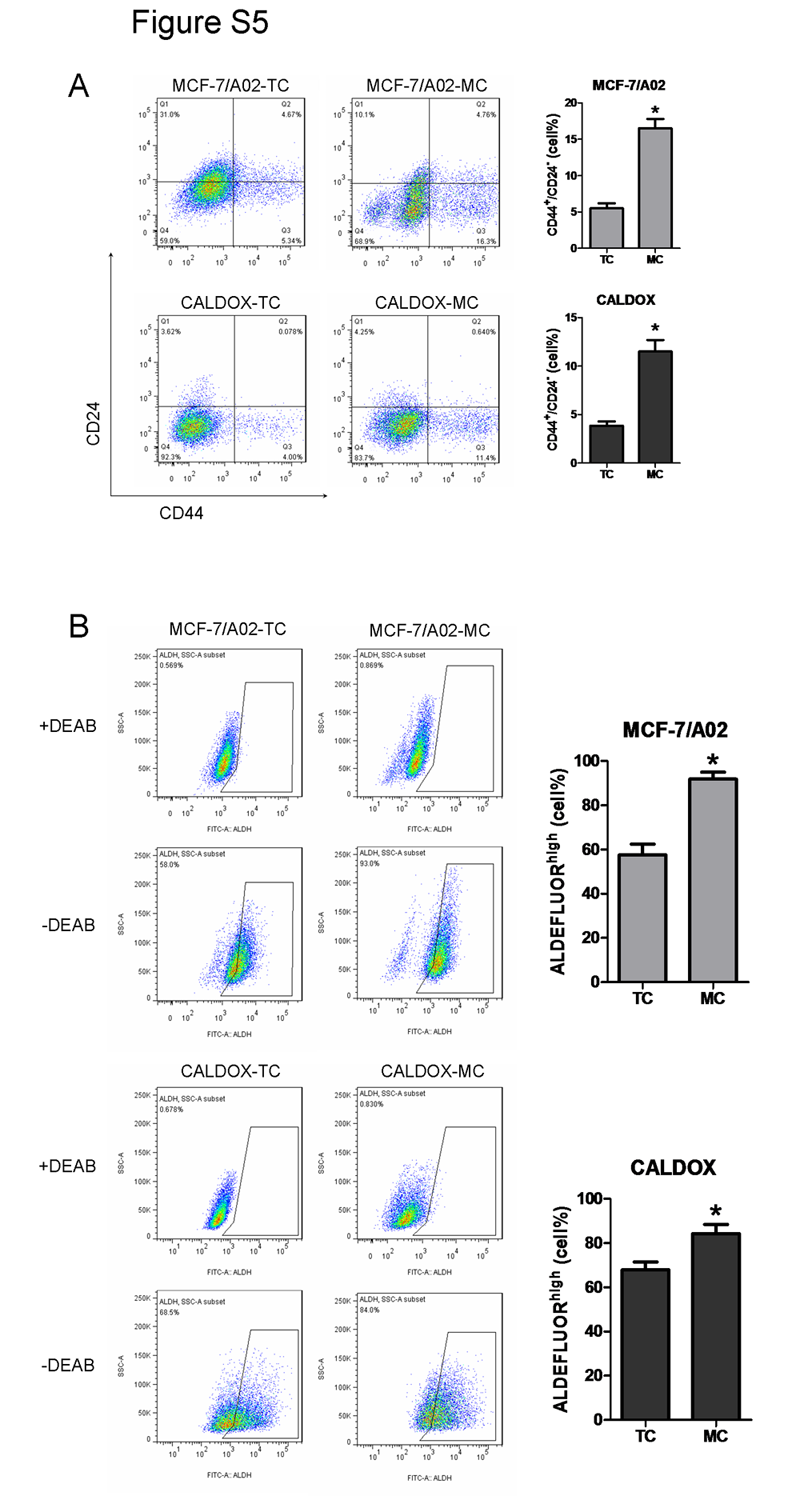


**Fig. S5. Mammosphere cells are rich of stem-like cells. (A)** Flow cytometry plots for CD44 and CD24 of total cells (TC) and mammosphere cells (MC) of MCF-7/A02 and CALDOX cells. Gating was set to unstained cells. Quantitative data show the average percentage of CD44^high^CD24^low^ cells (right panel) **(B)** ALDH activity of TC and MC analyzed by flow cytometry. Cells were assayed with an Aldefluor assay kit in the presence and absence of the ALDH inhibitor DEAB. Gating in the control was set up to a maximum of 1% of cells. Representative plots of at least three independent experiments are shown. Quantitative data show the average percentage of ALDH^high^ cells (right panel)


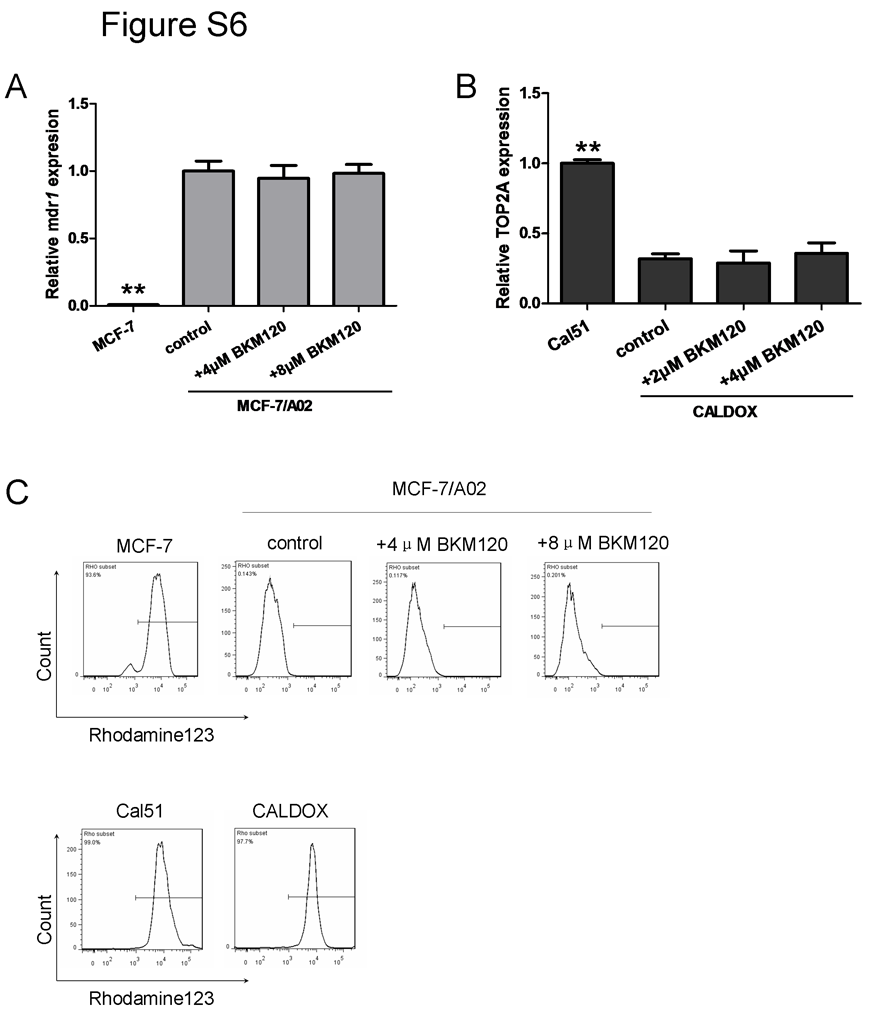


**Fig. S6. BKM120 does not affect mdr-1 or TOP2A expression. (A)** mdr-1 expression was determined by RT-qPCR and normalized with RPS14 in MCF-7/A02 cells treated with BKM120. Data are expressed as fold difference relative to non-treated control and presented as mean ± SD of three independent experiments. **(B)** TOP2A expression was determined by RT-qPCR and normalized with the house-keeping gene RPS14 in CALDOX cells. Data are expressed as fold difference relative to non-treated control and presented as mean ± SD of three independent experiments. **(C)** Cellular Rhodamine 123 content was measured by flow cytometry. Percentage of cells with intracellular Rhodamine 123 accumulation (gate) is shown. Representative pictorial data of three independent replicates are shown.

**Table S1** MCF-7/A02 cell sensitivity to different drugs

| Drug | IC_50_ | | MCF-7/A02 resistance ratio |
| --- | --- | --- | --- |
|  | MCF-7 | MCF-7/A02 |  |
| Doxorubicin | 2.64μM | 128.3μM | 48.6 |
| Etoposide | 5.29μM | 206.8μM | 39.1 |
| Taxol | 4.77nM | >500nM | >100 |
| Mitoxantrone | 8.7μM | 161.8μM | 18.6 |

**Table S2** CALDOX cell sensitivity to different drugs

| Drug | IC_50_ | | CALDOX  resistance ratio |
| --- | --- | --- | --- |
|  | Cal51 | CALDOX |  |
| Doxorubicin | 0.14μM | 5.73μM | 40.9 |
| Etoposide | 0.78μM | 26.8μM | 34.4 |
| Taxol | 2.77nM | 8.68nM | 3.13 |
| Mitoxantrone | 2.49nM | >300nM | >120 |

**Table S3** Oligonucleotides used in this study

| Name | Sequence (5' to 3') | Use |
| --- | --- | --- |
| RPS14-forward | TCACCGCCCTACACATCAAACT | real-time PCR |
| RPS14-reverse | CTGCGAGTGCTGTCAGAGG | real-time PCR |
| bax-forward | CGAGTGGCAGCTGACATGTTTT | real-time PCR |
| bax-reverse | TGAGGCAGGTGAATCGCTTGAA | real-time PCR |
| bim-forward | GCCGCCACTACCACCACTT | real-time PCR |
| bim-reverse | AACCGAATACCGCGATGATG | real-time PCR |
| survivin-forward | GGACCACCGCATCTCTACAT | real-time PCR |
| survivin-reverse | GACAGAAAGGAAAGCGCAAC | real-time PCR |
| NF-κB-forward | GATTTCGTTTCCGTTATG | real-time PCR |
| NF-κB-reverse | TTTGCTGGTCCCACATAG | real-time PCR |
| mdr-1-forward | CCCATCATTGCAATAGCAGG | real-time PCR |
| mdr-1-reverse | GTTCAA ACTTCTGCTCCTGA | real-time PCR |
| TOP2A-forward | TCATCAAGATTGTGGGTCTTCAG | real-time PCR |
| TOP2A-reverse | CCTCCAGAAAACGATGTCGCA | real-time PCR |
